# Investigation on the mechanisms of carbapenem resistance among the non-carbapenemase-producing carbapenem-resistant *Klebsiella pneumoniae*

**DOI:** 10.3389/fcimb.2024.1464816

**Published:** 2024-09-18

**Authors:** Yee Qing Lee, Sasheela Sri La Sri Ponnampalavanar, Jia Haw Wong, Zhi Xian Kong, Soo Tein Ngoi, Rina Karunakaran, Min Yi Lau, Kartini Abdul Jabar, Cindy Shuan Ju Teh

**Affiliations:** ^1^ Department of Medical Microbiology, Faculty of Medicine, Universiti Malaya, Kuala Lumpur, Malaysia; ^2^ Department of Medicine, Faculty of Medicine, Universiti Malaya, Kuala Lumpur, Malaysia; ^3^ Department of Anaesthesiology, Faculty of Medicine, Universiti Malaya, Kuala Lumpur, Malaysia

**Keywords:** cation efflux system protein CusF, missense mutation, NC-CRKP, nickel/cobalt efflux protein RcnA, ompK36 porin

## Abstract

**Background:**

In Malaysia, an increase in non-carbapenemase-producing carbapenem-resistant *Klebsiella pneumoniae* (NC-CRKP) has been observed over the years. Previously, four NC-CRKP with increased susceptibility to ciprofloxacin in the presence of phenylalanine-arginine β-naphthylamide (PAβN) were identified. However, no contribution of the PAβN-inhibited efflux pump to carbapenem resistance was observed. All four NC-CRKP harboured non-carbapenemase β-lactamase, with two also exhibiting porin loss. In this study, we further investigated the genomic features and resistance mechanisms of these four isolates.

**Methods:**

All four NC-CRKP were subjected to whole-genome sequencing, followed by comparative genomic and phylogenetic analyses.

**Results:**

Multi-locus sequence typing (MLST) analysis divided the four NC-CRKP into different sequence types: ST392, ST45, ST14, and ST5947. Neither major nor rare carbapenemase genes were detected. Given the presence of non-carbapenemase β-lactamase in all isolates, we further investigated the potential mechanisms of resistance by identifying related chromosomal mutations. Deletion mutation was detected in the cation efflux system protein CusF. Insertion mutation was identified in the nickel/cobalt efflux protein RcnA. Missense mutation of ompK36 porin was detected in two isolates, while the loss of ompK36 porin was observed in another two isolates.

**Conclusions:**

This study revealed that NC-CRKP may confer carbapenem resistance through a combination of non-carbapenemase β-lactamase and potential chromosomal mutations including missense mutation or loss of ompK36 porin and/or a frameshift missense mutation in efflux pump systems, such as cation efflux system protein CusF and nickel/cobalt efflux protein RcnA. Our findings highlighted the significance of implementing whole-genome sequencing into clinical practice to promote the surveillance of carbapenem resistance mechanisms among NC-CRKP.

## 1 Introduction

Carbapenem-resistant Enterobacterales (CREs) have been listed as an urgent threat to public health. CRE has emerged as a challenge in healthcare settings due to the limited treatment options (CDC, 2019a). CREs are Enterobacterales that confer resistance to at least one of the carbapenem antibiotics or produce a carbapenemase enzyme (CDC, 2019b, 2015). Among CRE infections, carbapenem-resistant *Klebsiella pneumoniae* (CRKP) is the most critical pathogen which was listed in the World Health Organization (WHO) priority list of antibiotic-resistant bacteria (Tacconelli et al., 2018). Studies pertaining to CRKP in the past decades have mainly focused on carbapenemase-producing CRKP (C-CRKP), predominantly due to the rapid global dissemination of carbapenemases (Lee et al., 2016). Nonetheless, non-carbapenemase-producing carbapenem-resistant *Klebsiella pneumoniae* (NC-CRKP) is of huge clinical importance yet underestimated due to a lack of relevant research.

NC-CRKP does not produce carbapenemase but can exhibit resistance to carbapenems through a combination of chromosomal mutations (e.g., porin gene mutation, overproduction of efflux pump, and/or alterations in cell structure such as penicillin-binding protein) and acquired non-carbapenemase resistance mechanisms (acquisition or upregulation of a β-lactamase) (CDC, 2019b, 2015; Westblade, 2018). Although studies suggested that C-CRKP was more virulent than NC-CRKP (Goodman et al., 2016; Tamma et al., 2017; Richards et al., 2017), the mortality rate of NC-CRKP was high (Su et al., 2018) and almost similar to C-CRKP (Orsi et al., 2013). Therefore, the significance of the disease burden posed by NC-CRKP should not be overlooked.

In Malaysia, studies on NC-CRKP are scarce, but the limited evidence garnered from these studies has demonstrated great diversity and dynamicity of local CRKP populations (Lau et al., 2021; Kong et al., 2022; Lee et al., 2022; Zaidah et al., 2017; Zawawi et al., 2021; Yeow, 2020; Heng et al., 2019; Mohamed et al., 2018; Low et al., 2017). An increasing trend of NC-CRKP was observed in our hospital setting. The number of cases of NC-CRKP increased from one in 2013 to 17 cases in 2018, followed by a slight decline to 12 cases in 2019 (Lee et al., 2022). NC-CRKP may potentially emerge through *de novo* mutations or genetic reassortment within carbapenem-sensitive Enterobacterales under antimicrobial selective pressure (Marimuthu et al., 2019). Although the prevalence of NC-CRKP remained lower than C-CRKP in Malaysia, the increasing trend should not be ignored since the treatment for NC-CRKP may be different from C-CRKP infections as NC-CRKP employ different mechanisms for carbapenem resistance. In addition, the lack of epidemiological data and an unknown mechanism may pose significant challenges in treating NC-CRKP.

In our recent study, 54 NC-CRKP isolated from 2013 to 2019 in Malaysia have been studied (Lee et al., 2022). The study identified the loss of porins (46.3%, 25/54) and the presence of *AmpC* β-lactamase gene (*bla*_DHA_), along with other non-carbapenemase β-lactamase genes (*bla*_TEM_, *bla*_SHV_, *bla*_CTX-M_, and *bla*_OXA-1_) in the NC-CRKP isolates. In addition, a minimum inhibitory concentration (MIC) reduction assay using 26.3 µg/ml phenylalanine-arginine β-naphthylamide (PAβN), a Resistance Nodulation Division (RND) efflux pump inhibitor (Dupont et al., 2016), was performed to address conflicting findings from previous studies regarding whether PAβN-inhibited efflux pumps reduce carbapenem MIC value by 1 to 4 times (Khalid and Ghaima, 2022) or elevate resistance to carbapenem antibiotics (Saw et al., 2016). While all NC-CRKP isolates demonstrated the same MIC value for ciprofloxacin, four isolates exhibited increased susceptibility to ciprofloxacin but also showed increased resistance to carbapenem (Lee et al., 2022). In this study, we sought to investigate the genomic features, carbapenem resistance mechanisms, and virulence determinants of these four NC-CRKP isolates, collected from a tertiary teaching hospital in 2018, using whole-genome sequencing (WGS) to shed light on the mechanisms of carbapenem resistance in NC-CRKP isolates attributable to potential chromosomal mutations.

## 2 Materials and methods

### 2.1 Study setting and clinical isolates

NC-CRKP was classified based on the absence of the five major carbapenemase genes, which were *bla*_NDM_, *bla*_OXA-48_, *bla*_IMP_, *bla*_VIM_, and *bla*_KPC_. In this study, four NC-CRKP isolated from the Universiti Malaya Medical Centre (UMMC) patients by the hospital’s Medical Microbiology Diagnostic Laboratory (MMDL) in 2018, designated 1801-2, 1804-1, 1805-11, and 1805-12 were selected and subjected to whole-genome sequencing (WGS) assays. Their clinical and antimicrobial characteristics have been previously described (Lee et al., 2022).

The rationale of selection was based on the presence of *AmpC* β-lactamase gene (*bla*_DHA_), other non-carbapenemase β-lactamase genes (*bla*_TEM_, *bla*_SHV_, *bla*_CTX-M_, and *bla*_OXA-1_), porins loss, and findings from a minimum inhibitory concentration (MIC) reduction assay conducted in a previous study (Lee et al., 2022). All four isolates harboured non-carbapenemase β-lactamase genes: *bla*_SHV_ was present in all four isolates; *bla*_TEM_ and *bla*_CTX-M_ were found in two isolates (1804-1 and 1805-11); and *bla*_OXA-1_ was detected in one isolate (1801-2). Two NC-CRKP isolates harboured the *AmpC* β-lactamase gene (*bla*_DHA_; 1804-1 and 1805-12), while the other two exhibited ompK36 porin loss (1801-2 and 1805-11). Two NC-CRKP isolates were mono-resistant to ertapenem (isolates 1801-2 and 1804-1), while the other two were resistant to all three carbapenems (1805-11 and 1805-12). The MIC reduction assay using the RND efflux pump inhibitor PAβN (26.3 µg/ml) revealed a ≥ 4-fold decrease in the MIC of ciprofloxacin in all four isolates (**Supplementary Table S1**) (Lee et al., 2022; Dupont et al., 2016). This implied the involvement of PAβN-inhibited efflux pumps in mediating ciprofloxacin resistance among these isolates. However, no active efflux contribution of PAβN-inhibited efflux pumps to carbapenem resistance was observed, suggesting diversity in the resistance mechanisms governing different antimicrobial classes among these four NC-CRKP isolates.

### 2.2 Genome sequencing, assembly, and annotation

Whole-genomic deoxyribonucleic acid (DNA) of the isolates was extracted using the DNeasy blood and tissue kit (Qiagen, Hilden, Germany) according to the manufacturer’s instructions. DNA concentrations were measured with a Qubit fluorometer to determine DNA input from each isolate. The quality of genomic DNA was assessed by agarose gel electrophoresis. Implen NanoPhotometer^®^ spectrophotometer (Implen GmbH, Munchen, Germany) was used to quantify the purity of the DNA samples.

WGS was performed using the Miseq platform using V3 reagent kits with paired-end 2x300 bp reads (Illumina, San Diego, CA, USA). Libraries were prepared by following the Illumina Nextera XT sample preparation guide. Sequences for individual isolates were demultiplexed by a local run manager (LRM) version 3.0.0. The raw data was pre-processed by trimming away poor-quality ends of the reads and poor-quality reads using Trimmomatic settings (Bolger et al., 2014) with SLIDINGWINDOW:4:20 and MINLEN:30 as trimming parameters. Clean data obtained after trimming was mapped to the *K. pneumoniae* subspecies *pneumoniae* HS11286 reference genome ASM24018v2 (NCBI RefSeq assembly: GCF_000240185.1). Variant calling was performed on the mapped data.

The raw sequencing reads in FASTQ format were uploaded to CLC Genomics Workbench version 7.5.1 (CLC bio, Aarhus C, Denmark). Sequence reads were trimmed by removing ambiguous nucleotides and those with Phred scores of < 30. *De novo* assembly was performed for each isolate with scaffolding, 25 bp word size, bubble size of 50 bp, and discarding contigs of < 250 bp. Contigs with low coverage (< 30.0% of the average genome coverage) were also removed from the genome assembly. Contigs with an average genome coverage of 30.0% and above were selected to represent the draft genome for further analysis.

The FASTA format of the assembled genomic sequence was uploaded to the web server of the Rapid Annotation using Subsystem Technology (RAST) at https://rast.nmpdr.org/for genomic annotation based on the National Center for Biotechnology Information (NCBI) taxonomy identifier 573 (NCBI:txid573) (Aziz et al., 2008; Overbeek et al., 2014; Brettin et al., 2015).

### 2.3 Whole genome sequence analyses

#### 2.3.1 Antimicrobial resistance genes prediction

The annotated reads were submitted to the Resistance Gene Identifier (RGI) version 6.0.1 to predict antimicrobial resistome from protein or nucleotide data by using curated reference data from the Comprehensive Antibiotic Resistance Database (CARD) version 3.2.6 (Alcock et al., 2023). Stringent selection criteria of antimicrobial resistance genes (ARG) were applied, filtering to only perfect (100.0% identity) and strict (CARD’s curated bit-score cut-offs) hits against the curated reference sequences in CARD.

The RGI predictions were cross-validated using ResFinder version 4.1 with a minimal threshold of 90.0% sequence identity and minimal resistance gene length coverage of 60.0% (Zankari et al., 2012).

The resistome was defined by consolidating the antimicrobial resistance gene predictions by both databases. All predicted ARG were validated through comprehensive cross-examination with RAST-annotation output and manual interrogation using NCBI Basic Local Alignment Search Tool (BLAST: blastx).

#### 2.3.2 Multi-locus sequence typing

The assembled genome was uploaded to Pasteur multi-locus sequence typing (MLST) version 2.0.9 of the Center for Genomic Epidemiology (CGE) web server (https://cge.food.dtu.dk/services/MLST/) to identify the sequence type (ST) of the studied isolates (Larsen et al., 2012). The PubMLST typing scheme assessing the seven conventional housekeeping genes (*gapA*, *infB*, *mdh*, *pgi*, *phoE*, *rpoB*, and *tonB*) was adopted for diversity characterization (Jolley et al., 2018).

#### 2.3.3 Identification of virulence genes

The draft genomes in Genbank format were uploaded to the VFanalyzer (http://www.mgc.ac.cn/cgi-bin/VFs/v5/main.cgi?func=VFanalyzer) for identification of virulence genes based on pre-analyzed reference genomes from the virulence factor database (VFDB).

#### 2.3.4 Identification of plasmids

The PlasmidFinder 2.1 (https://cge.food.dtu.dk/services/PlasmidFinder/) database was used to search for potential plasmid sequences in the draft genomes (Carattoli et al., 2014). The mapping criteria were set at ≥ 95.0% sequence identity and ≥ 60.0% mutual coverage to a reference plasmid (Carattoli et al., 2014).

#### 2.3.5 Prediction of carbapenem resistance mechanism in NC-CRKP

To identify potential chromosomal mutations contributing to carbapenem resistance, RAST-annotated gene sequences associated with peptidoglycan biosynthesis, peptidoglycan maturation, and the efflux pump system were compared with the corresponding NCBI reference sequences within the BioCyc databases (Karp et al., 2019) available at http://BioCyc.org. The pairwise comparison was performed in MEGA11: Molecular Evolutionary Genetics Analysis version 11 (Tamura et al., 2021). Furthermore, porin-associated genes (*ompK35* and *ompK36*) were amplified via polymerase chain reaction (PCR) (Kaczmarek et al., 2006). The PCR amplicons were sequenced and compared with curated gene sequences in the GenBank using the NCBI BLAST. Specifically, the ompK35 porin was aligned with the wild-type ompK35 porin (GenBank accession number AJ011501) (Doménech-Sánchez et al., 2003), while the ompK36 porin was aligned with the wild-type ompK36 porin (GenBank accession number Z33506) (Albertí et al., 1995).

## 3 Results

### 3.1 Genome sequences features

In 2018, a total of 65 CRKP isolates were collected from patients’ clinical and screening samples. Of these, 26.2% were classified as NC-CRKP, while the remaining isolates were identified as C-CRKP because they harboured carbapenemase genes. The colonization rate among NC-CRKP patients was 64.7% (11/17) in 2018.

Four NC-CRKP isolates, designated 1801-2, 1804-1, 1805-11, and 1805-12, were previously isolated from rectal swabs of UMMC patients during routine screening in 2018. All isolates were confirmed as colonizers. Only 1 patient (1801-2) was associated with all-cause in-hospital mortality. These four NC-CRKP isolates were subjected to whole-genome sequencing. The sequences were analyzed for their virulence and potential determinants of resistance mechanisms. In brief, the size of the genomes ranged from 5.4 to 5.7 million base pairs (Mbp), with an approximate GC content of 57.0%. The draft genome was consistent with the typical *K. pneumoniae* genome, which is approximately 5.5 Mbp (Wyres et al., 2020). The genomic features of these four NC-CRKP isolates are summarized in **Table 1**. All the draft genomes have been deposited in the NCBI GenBank with BioSample accession of SAMN41156152 (1801-2), SAMN41156273 (1804-1), SAMN41156274 (1805-11), and SAMN41156276 (1805-12).

**Table 1:** General genome features of the NC-CRKP isolates.

Isolates	1801-2	1804-1	1805-11	1805-12
Genome size (bp)	5,536,202	5,716,583	5,542,162	5,429,419
Total genome coverage	104 x	100 x	93 x	79 x
N50 (bp)	355,343	358,427	364,263	350,629
GC content (%)	57.2	57.1	57.3	57.2
Contig count	83	86	74	74
Number of CDS	5512	5745	5432	5367
Number of RNA	89	88	84	88

The antimicrobial characteristics of the four NC-CRKP isolates, including the susceptibility profile, resistance genes, and porin-associated genes, were summarized in **Table 2**. Two of the *Klebsiella pneumoniae* isolates, 1805-11 and 1805-12 exhibited resistance to all tested carbapenems (one with porin loss and another with porin mutation), while the other two, 1801-2 and 1804-1, were mono-resistant to ertapenem only (one with porin loss and another with porin mutation). The identified bacterial species and resistance genes were concordant with laboratory data. None of the NC-CRKP harboured the five major carbapenemase genes.

**Table 2:** Antimicrobial characteristics by isolate.

Isolates	Antimicrobial class	Antimicrobial resistance genes (ARG)
**1801-2** ^*,a^	β-lactam:	*bla*_SHV-11_, *bla*_OXA-1_
	Folate pathway antagonist:	*sul3, dfrA12*
	Aminoglycosides:	*aadA2, AAC(3)-IV, APH(4)-Ia*
	Quinolone:	*QnrB1*
**1804-1** ^†,b^	β-lactam:	*bla*_SHV-1_, *bla*_TEM-1_, *bla*_CTX-M-15_, *bla*_DHA-1_
	Folate pathway antagonist:	*sul1, sul2, sul3, dfrA12*
	Aminoglycosides:	*aadA2*
	Quinolone:	*QnrB4, QnrS1*
**1805-11** ^*,b,c^	β-lactam:	*bla*_SHV-28_, *bla*_TEM-1_, *bla*_CTX-M-15_
	Folate pathway antagonist:	*sul1, dfrA27*
	Quinolone:	*QnrB6*
	Rifamycin:	*arr-3*
	Macrolide:	*mphA*
	Quaternary ammonium compound:	*qacE*
**1805-12** ^†,b,c^	β-lactam:	*bla*_SHV-1_, *bla*_DHA-1_
	Folate pathway antagonist:	*sul1, sul3, dfrA12*
	Aminoglycosides:	*aadA2*
	Quinolone:	*QnrB4, QnrS1*
	Macrolide:	*mphA, mef(B)*

^*^Loss of *ompK36* gene.

^†^Missense mutation in ompK36 porin.

a Isolate resistant to gentamicin (aminoglycosides).

b Isolate resistant to ceftazidime and ceftriaxone.

c Isolate resistant to imipenem and meropenem.

All four NC-CRKP isolates were found to possess ompK35 and ompK37 porins. All isolates were resistant to amoxicillin/clavulanate, ampicillin, cefuroxime, cefotaxime, ciprofloxacin (fluoroquinolones), and ertapenem.

### 3.2 Whole genome sequence analyses

#### 3.2.1 Antimicrobial resistance genes prediction

Antimicrobial resistance genes that confer resistance to different classes of antimicrobial agents were determined. As a gentamicin-resistant strain, isolate 1801-2 harboured aminoglycoside *N*-acetyltransferase 3-IV (*AAC(3)-IV*) and aminoglycosides *O*-phosphotransferases 4-Ia (*APH(4)-Ia*), which encodes resistant to apramycin, gentamicin, netilmicin, and tobramycin (Hao et al., 2020). Isolate 1805-11 harboured plasmid-encoded disinfectant resistance gene *qacE* which is associated with quaternary ammonium compound. This finding is worrying as the quaternary ammonium compound is widely present in cleaning, disinfecting, and personal care products.

In this study, NC-CRKP carried the non-carbapenemase β-lactamase gene that can confer resistance to β-lactam antibiotics, including plasmid-mediated *AmpC* β-lactamase gene such as *bla*_DHA-1_; extended-spectrum β-lactamase (ESBL) genes such as *bla*_CTX-M-15_ and *bla*_SHV-28_; narrow-spectrum class D β-lactamase gene such as *bla*_OXA-1_; and narrow-spectrum class A β-lactamase genes such as *bla*_TEM-1_, *bla*_SHV-1_ and *bla*_SHV-11_ (non-ESBL) (Bush and Jacoby, 2010; Fisher et al., 2005; Jones et al., 2018). None of the major and rare carbapenemase genes (*bla*_NDM_, *bla*_OXA-48_, *bla*_IMP_, *bla*_VIM_, *bla*_KPC_, *bla*_OXA-372_, *bla*_IMI_, *bla*_NMC_, *bla*_FRI_, *bla*_GES_, *bla*_BKC_, *bla*_SFC_, *bla*_SME_, *bla*_GIM_, *bla*_TMB_, *bla*_LMB_, *bla*_KHM_, *bla*_SFH_, *bla*_AIM_, *bla*_CMY_, *bla*_ACT_, or *bla*_BIC_) were detected among four NC-CRKP isolates. The antimicrobial resistance gene profiles were categorized by antimicrobial class in **Table 2**.

The acquisition of dihydropteroate synthase (DHPS) genes (*sul1*, *sul2*, and *sul3*) in integrons can contribute to sulfonamide (e.g., sulfamethoxazole) resistance (Gündoğdu et al., 2011), while *dfrA12* and *dfrA27* can confer resistance to trimethoprim (Ambrose and Hall, 2021), both hindering the folate biosynthesis pathway and contributing resistance against folate pathway inhibitors (Shin et al., 2015). The aminoglycoside-modifying enzyme (AME) genes mediate resistance to aminoglycoside antibiotics and compromise their efficacy in protein synthesis inhibition (Krause et al., 2016). The presence of a transferable target protection mechanism of quinolone resistance (*QnrB1*, *QnrB4*, *QnrB6*, and *QnrS1*) can protect bacterial DNA gyrase and topoisomerase IV from inhibition, thereby impeding bacterial DNA replication and reducing the effectiveness of treatments against bacterial infections (Ruiz, 2019). Rifampicin resistance may arise from the horizontal acquisition of ADP-ribosyltransferases such as *arr-3* that can inactivate rifampicin and other rifamycin (Fonseca et al., 2008; Morgado et al., 2021). The *mphA* and *mef(B)* may confer resistance to macrolides, such as erythromycin, azithromycin, spiramycin, and telithromycin, limiting their efficacy in inhibiting bacterial protein synthesis (Gomes et al., 2017; Katz and Ashley, 2005).

#### 3.2.2 Multi-locus sequence typing

The ST varied among the four NC-CRKP isolates. Isolates 1801-2, 1804-1, 1805-11, and 1805-12 were identified as ST392, ST45, ST14, and ST5947 respectively.

#### 3.2.3 Identification of virulence genes

The examination of the virulence factor (VF) of the four NC-CRKP isolates was crucial in elucidating their potential pathogenicity. Within the VF class of adherence, specific VF included type 1 fimbriae, type 3 fimbriae, and type IV pili. All isolates exhibited the presence of type 3 fimbriae genes (*mrkA*, *mrkB*, *mrkC*, *mrkD*, *mrkF*, *mrkH*, *mrkI*, *mrkJ*) and type 1 fimbriae genes (*fimA*, *fimB*, *fimC*, *fimD*, *fimE*, *fimF*, *fimG*, *fimH*, *fimI*, *fimK*). The gene *pilW*, associated with type IV pili, was not detected in isolate 1804-1, but it was identified in the remaining three isolates. The absence of *pilW* may indicate alternative type IV pili-related adhesion strategies or variations in pathogenicity.

In terms of antiphagocytosis, all isolates exhibited the presence of capsule-associated genes, and they were varied in the components essential to produce protective capsules or the structure of antiphagocytic barriers. While for the efflux pump, the presence of the AcrAB efflux pump system (*acrA* and *acrB* genes) was detected in all isolates.

Within the VF class of iron uptake mechanism, specific VF such as aerobactin, enterobactin (ent) siderophore, salmochelin, and yersiniabactin were detected. These VF are essential for acquiring iron, a vital nutrient for bacterial growth and survival (Foley and Simeonov, 2012). All isolates showed the presence of *iutA*, which is responsible for the expression of a specific outer membrane receptor protein for ferric aerobactin (Ferreira et al., 2016). The *fepA*, *fepB*, *fepC*, *fepD*, *fepG*, and *fes* (cytoplasmic ent esterase) required for the import of enterobactin were identified in all isolates. For the export of enterobactin, the *entA*, *entB*, *entC*, *entD*, *entE*, and *entS* (ent exporter gene) were identified in all isolates. The *entF* was identified in three isolates, except for isolate 1805-11. To produce salmochelin, the synthesis of enterobactin and the *iroBCDEN* gene cluster are essential (Zhu et al., 2005). The *iroE* (periplasmic esterase) and *iroN* (outer membrane receptor for salmochelin) were identified in all isolates, whereas the *iroB* (C-glucosyltransferase), *iroC* (an ABC-transporter for the uptake of salmochelin), and *iroD* (cytoplasmic esterase) were not found. In addition, the yersiniabactin peptide synthetase (e.g., *irp1*, *irp2*), salicyl-AMP ligase (*ybtE*), salicylate synthesis (*ybtS*), outer membrane protein (*fyuA*), transcriptional regulator (*ybtA*), reductase (*ybtU*), inner membrane permease for yersiniabactin uptake (e.g., *ybtP*, *ybtQ*), thioesterase (*ybtT*), and inner membrane transporter (*ybtX*) were identified in 1804-1 and 1805-11 isolates.

Within the VF class of regulation, the presence of *rcsA* and *rcsB* in RcsAB (regulator of capsule synthesis) system was detected in all isolates. For the VF class of secretion system, the genes in type VI secretion system cluster 1 (T6SS-I) and 3 (T6SS-III) were identified in all isolates. For instance, the genes found in T6SS-I were *clpV/tssH*, *dotU/tssL*, *hcp/tssD*, *icmF/tssM*, *ompA*, *sciN/tssJ*, *tssF*, *tssG*, *vasE/tssK*, *vgrG/tssI*, *vipA/tssB*, *vipB/tssC*, and *clpV*, while the genes in T6SS-III such as *dotU*, *impA*, *impF*, *impG*, *impH*, *impJ*, *ompA*, *sciN*, and *vgrG* were detected.

In the VF class of serum resistance, the VF-associated gene of lipopolysaccharide (LPS) biosynthetic (*rfb*) locus was detected in 1804-1, 1805-11, and 1805-12 isolates. In terms of autotransporter, the isolates 1805-11 and 1805-12 were also detected with the *cah* gene of calcium-binding antigen homologue (cah), which is involved in autoaggregation and biofilm formation.

#### 3.2.4 Identification of plasmids

Based on the analysis, only isolates 1801-2 and 1805-12 harboured plasmid of the incompatibility (Inc) R group, i.e., *IncR* type plasmid (GenBank accession number DQ449578), with 100.0% identity and coverage. Isolate 1804-1 harboured *IncR* plasmid with an identity of 99.2% and 100.0% coverage. No *IncR* plasmid was detected in isolate 1805-11.

#### 3.2.5 Prediction of carbapenem resistance mechanism in NC-CRKP

NC-CRKP confers carbapenem resistance through a combination of chromosomal mutations (e.g., alteration in cell structure, efflux pump, and/or porin) and acquired non-carbapenemase resistance mechanisms (acquisition or upregulation of a β-lactamase) (CDC, 2015, 2019; Westblade, 2018; Lee et al., 2022, 2020). Given the presence of multiple non-carbapenemase β-lactamase genes in all four NC-CRKP isolates, our study focused on elucidating the potential mechanisms of resistance by identifying related chromosomal mutations. Based on the limited cases identified, it has been suggested that NC-CRKP emerges due to reduced outer membrane permeability and/or increased drug efflux (Rosas et al., 2023; Goodman et al., 2016). Therefore, detailed analyses of all three aspects, including cell structure, efflux pump, and porin, revealed potential chromosomal mutations that could lead to carbapenem resistance in NC-CRKP.

A thorough analysis of both the peptidoglycan biosynthesis I pathway and the peptidoglycan maturation pathway of *Klebsiella pneumoniae* was conducted to discern alterations in cell structure (**Supplementary Table S2**). All 12 enzymes required for the peptidoglycan biosynthesis I pathway and all 17 enzymes required for the peptidoglycan maturation pathway were identified in all isolates. No alteration in the biosynthesis of cell structure was observed in isolate 1805-11. For the remaining isolates, amino acid substitutions or polymorphisms were identified in 58.3% (7/12) and 58.8% (10/17) of the enzymes required for the peptidoglycan biosynthesis I pathway and the peptidoglycan maturation pathway respectively.

On the other hand, a comprehensive examination of efflux genes, encompassing both polypeptide and transporter components, was performed among four NC-CRKP isolates to ascertain modifications in the efflux pump system. There were 78 efflux proteins examined in this study. Of the 71 efflux proteins detected in all isolates, 25 showed 100.0% identity, while 46 exhibited amino acid substitutions or polymorphisms. The presence of the remaining seven efflux proteins was accentuated in **Table 3**.

**Table 3:** Efflux proteins of *Klebsiella pneumoniae*.

Gene	Enzyme	Accession number	1801-2	1804-1	1805-11	1805-12
*A8C11_RS00185/A8C11_RS00810*	Quaternary ammonium compound efflux SMR transporter QacE delta 1	WP_000679427.1	Yes^†^	Yes^†^	–	Yes^†^
*A8C11_RS25050*	DHA2 family efflux MFS transporter permease subunit	WP_004179966.1	Yes^†^	–	Yes	–
*rfbB*	O-antigen export ABC transporter ATP-binding protein RfbB	WP_002912371.1	Yes^*^	–	Yes	Yes^†^
*rfbA*	O-antigen export ABC transporter permease RfbA	WP_002912373.1	Yes^*^	Yes^*^	Yes	Yes
*A8C11_RS18380*	efflux RND transporter periplasmic adaptor subunit	WP_004178902.1	Yes^†^	Yes^*,a^	Yes	Yes^†^
*A8C11_RS17640*	nickel/cobalt efflux protein RcnA	WP_043906842.1	Yes^†^	Yes^*,b^	Yes^*,c^	Yes^*,d^
*cusF*	cation efflux system protein CusF	WP_004197273.1	Yes^*,e^	Yes^†^	Yes	Yes^†^

^*^Isolate exhibited missense mutations in efflux proteins.

^†^Isolate exhibited amino acid substitutions or polymorphisms in efflux proteins.

a Isolate exhibited an insertion mutation of QQQ between positions 327 and 328.

b Isolate exhibited an insertion mutation of RHDHDH between positions 145 and 146.

c Isolate exhibited an insertion mutation of HYHEHD between positions 138 and 139.

d Isolate exhibited an insertion mutation of PEHDHHPEHDHH between positions 146 and 147.

e Isolate exhibited a deletion mutation of HAGMAMHEEPAAA between positions 26 and 38.

The symbol “-” denotes the absence of an enzyme.

**Figure 1:**
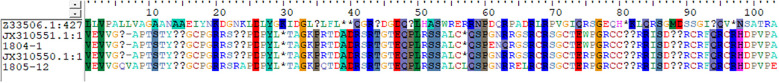
Missense mutation in ompK36 porin of NC-CRKP. The identities and similarities were shaded in the alignment window.

A pairwise comparison of porin-associated genes with the wild-type porin was performed to identify and characterize any potential mutations in the porin genes. All four NC-CRKP isolates were found to possess a wild-type ompK35 porin, as previously documented (GenBank accession number AJ011501). However, these isolates exhibited silent mutations in *ompK35* (**Table 4**). Isolates 1801-2 and 1805-11 exhibited ompK36 porin loss. Notably, isolates 1804-1 and 1805-12 exhibited missense mutations in ompK36 porin, establishing a closer genetic relationship to JX310551.1 and JX310550.1, respectively, which belong to the ST11 lineage of *bla*_KPC-2_-producing *K. pneumoniae* from China (**Figure 1**) (Zhang et al., 2014).

**Table 4:** Silent mutations in *ompK35* of NC-CRKP.

Isolate	Position					
	303	420	474	537	648	786
AJ011501.1	A	C	T	C	C	C
1801-2	–	T	C	T	–	T
1804-1	–	–	–	T	G	–
1805-11	G	–	C	–	–	T
1805-12	G	T	C	–	–	T

All isolates exhibited silent mutations in *ompK35* compared to the wild-type ompK35 porin (AJ011501). The silent mutations occurred at different positions in each isolate. The symbol “-” denotes no mutation occurred at that position.

## 4 Discussion

The emergence of NC-CRKP has posed a significant challenge in healthcare settings (Hovan et al., 2021). The absence of major carbapenemase genes, such as *bla*_NDM_, *bla*_OXA-48_, *bla*_IMP_, *bla*_VIM_, and *bla*_KPC_, in NC-CRKP emphasized the importance of exploring the other resistance mechanisms acquired by non-carbapenemase-producing carbapenem-resistant isolates. Efflux pumps or porins in combination with β-lactamase genes, have been identified as potential contributors to carbapenem resistance in *Klebsiella pneumoniae* (Nicolas-Chanoine et al., 2018). Nicolas-Chanoine et al. (2018) reported that *bla*_DHA-1_-producing isolates conferred resistance to ertapenem when there was an increase in efflux or porin loss, and to imipenem when both mechanisms were associated. In our study, both isolates 1804-1 (mono-resistant to ertapenem) and 1805-12 (resistant to all three carbapenems) harbouring *bla*_DHA-1_ did not have porin loss but exhibited a missense mutation in the ompK36 porin and a frameshift insertion mutation in efflux protein. Furthermore, Nicolas-Chanoine et al. (2018) also found that *bla*_CTX-M-15_-producing isolates developed resistance to ertapenem in the absence of porins. In our study, isolates 1804-1 and 1805-11 harbouring *bla*_CTX-M-15_ also exhibited a frameshift insertion mutation in efflux protein. Only isolate 1805-11 (resistant to all three carbapenems) exhibited ompK36 porin loss, whereas isolate 1804-1 (mono-resistant to ertapenem) exhibited a missense mutation in ompK36 porin but no porin loss. Additionally, previous studies have presented conflicting results on whether PAβN-inhibited efflux pumps reduce carbapenem MIC value by 1 to 4 times (Khalid and Ghaima, 2022) or elevate resistance to carbapenem antibiotics (Saw et al., 2016). To address this controversy, a MIC reduction assay was conducted using the RND efflux pump inhibitor PAβN on 54 NC-CRKP isolates (Lee et al., 2022). Our results concurred with Saw et al. (2016), indicating that the PAβN-inhibited efflux pump did not contribute to carbapenem resistance although increased susceptibility to ciprofloxacin.

Throughout the years, ST101 has been identified as the predominant sequence type among C-CRKP in Malaysia (Low et al., 2017). This is in concordance with other countries such as Italy (Mammina et al., 2012) and Spain (Fuster et al., 2020). However, none of the isolates in this study were of ST101. This suggested the genetic heterogeneity among the NC-CRKP isolates.

Besides the loss of porins as previously reported by Lee et al. (2022), this study further revealed the silent mutations of ompK35 porin in all isolates and the missense mutations in ompK36 porin. The loss or alteration of ompK36 porin has been associated with carbapenem resistance (Kaczmarek et al., 2006; Clancy et al., 2013).

This study has raised a few concerns. Firstly, the identification of *IncR* plasmids suggested the potential for horizontal gene transfer and dissemination of resistance determinants. The *IncR* plasmids detected in this study were identical to the plasmid pK245 (GenBank accession number DQ449578) that confers quinolone resistance and ESBL activity (Chen et al., 2006). *IncR* plasmids carry various antimicrobial resistance genes that mediate resistance to β-lactam, aminoglycoside, phenicol, tetracycline, sulfonamide, and trimethoprim (Schwanbeck et al., 2021).

Secondly, the presence of plasmid-encoded disinfectant resistance gene *qacE* identified in the isolate 1805-11 could reduce the disinfectant susceptibility and confer quaternary ammonium compound protection in *K. pneumoniae* isolates (Liu et al., 2024; Paulsen et al., 1993). A wide distribution of disinfectant resistance genes resulting from the widespread use of disinfectants could impose selective pressure on antimicrobial resistance isolates (Liu et al., 2024; Abuzaid et al., 2012). This was in accordance with a previous study that suggested that NC-CRKP was confined to individuals and settings with very high levels of antimicrobial selection pressure (Richards et al., 2017).

Thirdly, the examination of virulence determinants according to the VF class highlighted potential pathogenicity in NC-CRKP, including adherence, antiphagocytosis, efflux pumps, iron uptake mechanisms, regulation, secretion system, serum resistance, and autotransporter. The presence of these VF suggested the ability of NC-CRKP to evade host defenses and establish infections. In this study, all NC-CRKP isolates were resistant to ciprofloxacin and carried the AcrAB efflux pump. As AcrAB multidrug efflux system is crucial for expelling harmful substances from the bacterial cellular environment, it may also influence the isolates’ resistance to antimicrobial agents and other toxic compounds (Padilla et al., 2010).

In this study, a few mutations identified could play important roles in resistance mechanisms. A frameshift deletion mutation was identified in the cation efflux system protein CusF (*cusF*) in isolate 1801-2. The cation efflux system protein CusF, also known as copper-binding periplasmic protein CusF, is present in the periplasm. It binds and transports periplasmic metal cations such as copper (Cu; Cuprum) or silver (Ag; Argentum) to the Cus (Cu sensing) system for efflux (Loftin et al., 2005; Kim et al., 2011; Franke et al., 2003; Lee and Choi, 2020). In previous studies, the periplasmic metallochaperone CusF demonstrated upregulated expression under the induction of silver cation (Imran et al., 2024) and copper cation in both aerobic and anaerobic conditions (Zulfiqar and Shakoori, 2012; Egler et al., 2005). The protein CusF utilizes cation and methionine interactions to bind Cu^+^ or Ag^+^ for delivery to the Cus system, as determined by the site-directed mutagenesis of methionine residue to isoleucine (substitution of methionine residue) or NMR chemical shift analysis (Loftin et al., 2005; Franke et al., 2003; Randall et al., 2015). We deduced that the frameshift deletion mutation in the cation efflux system protein CusF, which particularly affects the methionine residues, as shown in our study, could lead to a conformational change in protein CusF, thus leading to the accumulation of copper ions in bacteria. Previous studies reported that copper ions may form complexes with meropenem, leading to meropenem structure degradation and thereby contributing to carbapenem resistance (Božić et al., 2018; Božić Cvijan et al., 2023). Metal ions may diminish the efficiency of antimicrobial activity through respective efflux pumps by forming complexes, that eventually may lead to the degradation of antimicrobial agents. Further investigation can be performed to explore the potential interaction between metal ions and antimicrobial agents with their impact on the efficiency of antimicrobial therapy.

Previously, Perron et al. (2004) reported that a co-increase in resistance to heavy metals and imipenem, along with the suppression of OprD porin, was associated with the overexpression of a mutated czc efflux system in *Pseudomonas aeruginosa* (Perron et al., 2004). This czc (cobalt/zinc/cadmium) efflux system is similar to the nickel/cobalt efflux protein RcnA in terms of the function where both efflux protein/system can export cobalt cations out of the cytosol when excessive toxicity is detected. In the present study, frameshift insertion mutations in the nickel/cobalt efflux protein RcnA (*A8C11_RS17640*) were identified in NC-CRKP (isolates 1804-1, 1805-11 and 1805-12). This mutation may potentially contribute to the carbapenem resistance, which is similar to the mechanism found in *Pseudomonas aeruginosa*. Additionally, the inactivation of *RcnA* (*yohM*), a nickel and cobalt resistance gene in *Escherichia coli*, conferred sensitivity to nickel and cobalt (Rodrigue et al., 2005). The *yohM* gene possesses a remarkable histidine-rich region composed of histidines, aspartate, and glutamate residues, which may function as nickel storage (Rodrigue et al., 2005). Rodrigue et al. (2005) reported that a mutant transformed with a high number of copies of vector harbouring *yohM* greatly enhanced the wild-type resistant levels by 100-fold (Rodrigue et al., 2005). In our study, a frameshift insertion mutation in the nickel/cobalt efflux protein RcnA (*A8C11_RS17640*) introduced additional histidine (H), aspartate (D), and glutamate (E) residues. This modification likely enhances the binding of metal ions by the histidine-rich region, potentially leading to an enhanced resistance level. This could enhance resistance towards imipenem, as described in the mechanism found in *Pseudomonas aeruginosa*. Nevertheless, future studies are warranted to confirm our hypothesis.

Based on the analysis, the potential mechanisms of carbapenem resistance in the four isolates were postulated: 1) Isolate 1801-2 may confer carbapenem resistance through ompK36 porin loss and efflux pump mutation (*cusF*) combined with the acquisition of non-ESBL (*bla*_SHV-11_ and *bla*_OXA-1_) genes. 2) The resistance of carbapenem in isolate 1805-11 may be due to the chromosomal mutations such as ompK36 porin loss and efflux pump mutation (*A8C11_RS17640*) in combination with the acquisition of non-ESBL (*bla*_TEM-1_) and ESBL (*bla*_CTX-M-15_ and *bla*_SHV-28_) genes. 3) A diverse combination of β-lactamase genes has been identified in isolate 1804-1 and could be coupled with ompK36 porin mutation and efflux pump mutation (*A8C11_RS17640*) for carbapenem resistance. 4) Isolate 1805-12 may confer carbapenem resistance through ompK36 porin mutation and efflux pump mutation (*A8C11_RS17640*) in combination with the acquisition of non-ESBL (*bla*_SHV-1_) and *AmpC* β-lactamase (*bla*_DHA-1_) genes. However, future studies are warranted in order to validate the genomic findings.

Based on the WGS data, we have identified potential contributing factors to carbapenem resistance mechanisms and elucidated the genomic characteristics as well as the virulence determinants of NC-CRKP isolates. Despite the small number of NC-CRKP isolates in this study, the relevance of our findings sheds light on this issue. To enhance the robustness of future studies, a larger sample size should be included to better assess the mechanisms of carbapenem resistance. Moreover, experimental confirmation through functional validation of the identified resistance genes and mutations is quintessential to confirm the roles of these genetic changes in resistance. Unraveling these potential mechanisms of carbapenem resistance and understanding the genetic heterogeneity within NC-CRKP isolates are imperative for devising effective strategies to control their dissemination and combat antimicrobial resistance in healthcare settings.

## Data Availability

The datasets presented in this study can be found in online repositories. The names of the repository/repositories and accession number(s) can be found in the article/**Supplementary Material**.

## References

[B1] Abuzaid A. Hamouda A. Amyes S. (2012). *Klebsiella pneumoniae* susceptibility to biocides and its association with *cepA*, *qacΔE* and *qacE* efflux pump genes and antibiotic resistance. J. Hosp. Infection 81, 87–91. doi: 10.1016/j.jhin.2012.03.003 22498639

[B2] Albertí S. Rodríquez-Quiñones F. Schirmer T. Rummel G. Tomás J. M. Rosenbusch J. P.. (1995). A porin from *Klebsiella pneumoniae*: Sequence homology, three-dimensional model, and complement binding. Infection Immun. 63, 903–910. doi: 10.1128/iai.63.3.903-910.1995 PMC173088 7868262

[B3] Alcock B. P. Huynh W. Chalil R. Smith K. W. Raphenya A. R. Wlodarski M. A.. (2023). CARD 2023: Expanded curation, support for machine learning, and resistome prediction at the Comprehensive Antibiotic Resistance Database. Nucleic Acids Res. 51, D690–D699. doi: 10.1093/nar/gkac920 36263822 PMC9825576

[B4] Ambrose S. J. Hall R. M. (2021). *dfrA* trimethoprim resistance genes found in Gram-negative bacteria: Compilation and unambiguous numbering. J. Antimicrobial Chemotherapy 76, 2748–2756. doi: 10.1093/jac/dkab212 34180526

[B5] Aziz R. K. Bartels D. Best A. A. DeJongh M. Disz T. Edwards R. A.. (2008). The RAST Server: Rapid annotations using subsystems technology. BMC Genomics 9, 1–15. doi: 10.1186/1471-2164-9-75 18261238 PMC2265698

[B6] Bolger A. M. Lohse M. Usadel B. (2014). Trimmomatic: A flexible trimmer for Illumina sequence data. Bioinformatics 30, 2114–2120. doi: 10.1093/bioinformatics/btu170 24695404 PMC4103590

[B7] Božić B. Korać J. Stanković D. M. Stanić M. Romanović M. Pristov J. B.. (2018). Coordination and redox interactions of β-lactam antibiotics with Cu2+ in physiological settings and the impact on antibacterial activity. Free Radical Biol. Med. 129, 279–285. doi: 10.1016/j.freeradbiomed.2018.09.038 30267756

[B8] Božić Cvijan B. Korać Jačić J. Bajčetić M. (2023). The impact of copper ions on the activity of antibiotic drugs. Molecules 28, 5133. doi: 10.3390/molecules28135133 37446795 PMC10343859

[B9] Brettin T. Davis J. J. Disz T. Edwards R. A. Gerdes S. Olsen G. J.. (2015). RASTtk: A modular and extensible implementation of the RAST algorithm for building custom annotation pipelines and annotating batches of genomes. Sci. Rep. 5, 8365. doi: 10.1038/srep08365 25666585 PMC4322359

[B10] Bush K. Jacoby G. A. (2010). Updated functional classification of β-lactamases. Antimicrobial Agents Chemotherapy 54, 969–976. doi: 10.1128/AAC.01009-09 19995920 PMC2825993

[B11] Carattoli A. Zankari E. Garcìa-Fernandez A. Larsen M. V. Lund O. Villa L.. (2014). PlasmidFinder and pMLST: In silico detection and typing of plasmids. Antimicrobial Agents Chemotherapy 58, 3895–3903. doi: 10.1128/AAC.02412-14 24777092 PMC4068535

[B12] CDC (2015). Facility guidance for control of carbapenem-resistant Enterobacteriaceae (CRE): November 2015 update - CRE toolkit (Atlanta: United States, Centers for Disease Control and Prevention).

[B13] CDC (2019a). Antibiotic resistance threats in the United State (Atlanta, GA: U.S. Department of Health and Human Services, CDC). Available at: https://www.cdc.gov/drugresistance/biggest-threats.html.

[B14] CDC (2019b). Carbapenem-resistant enterobacterales (CRE) (Atlanta, GA: US Department of Health and Human Services, Centers for Disease Control and Prevention). Available at: https://www.cdc.gov/hai/organisms/cre/technical-info.html.

[B15] Chen Y. T. Shu H. Y. Li L. H. Liao T. L. Wu K. M. Shiau Y. R.. (2006). Complete nucleotide sequence of pK245, a 98-kilobase plasmid conferring quinolone resistance and extended-spectrum-beta-lactamase activity in a clinical *Klebsiella pneumoniae* isolate. Antimicrobial Agents Chemotherapy 50, 3861–3866. doi: 10.1128/AAC.00456-06 16940067 PMC1635178

[B16] Clancy C. J. Chen L. Hong J. H. Cheng S. Hao B. Shields R. K.. (2013). Mutations of the *ompK36* porin gene and promoter impact responses of sequence type 258, *KPC-2*-producing *Klebsiella pneumoniae* strains to doripenem and doripenem-colistin. Antimicrobial Agents Chemotherapy 57, 5258–5265. doi: 10.1128/AAC.01069-13 23939888 PMC3811244

[B17] Doménech-Sánchez A. Martínez-Martínez L. Hernández-Allés S. del Carmen Conejo M. Pascual A. Tomás J. M.. (2003). Role of *Klebsiella pneumoniae* ompK35 porin in antimicrobial resistance. Antimicrobial Agents Chemotherapy 47, 3332–3335. doi: 10.1128/AAC.47.10.3332-3335.2003 14506051 PMC201126

[B18] Dupont H. Gaillot O. Goetgheluck A.-S. Plassart C. Emond J.-P. Lecuru M.. (2016). Molecular characterization of carbapenem-nonsusceptible enterobacterial isolates collected during a prospective interregional survey in France and susceptibility to the novel ceftazidime-avibactam and aztreonam-avibactam combinations. Antimicrobial Agents Chemotherapy 60, 215–221. doi: 10.1128/AAC.01559-15 26482307 PMC4704208

[B19] Egler M. Grosse C. Grass G. Nies D. H. (2005). Role of the extracytoplasmic function protein family sigma factor RpoE in metal resistance of *Escherichia coli* . J. Bacteriology 187, 2297–2307. doi: 10.1128/JB.187.7.2297-2307.2005 PMC1065229 15774872

[B20] Ferreira D. Seca A. M. Diana C. Silva A. M. (2016). Targeting human pathogenic bacteria by siderophores: A proteomics review. J. Proteomics 145, 153–166. doi: 10.1016/j.jprot.2016.04.006 27109355

[B21] Fisher J. F. Meroueh S. O. Mobashery S. (2005). Bacterial resistance to β-lactam antibiotics: Compelling opportunism, compelling opportunity. Chem. Rev. 105, 395–424. doi: 10.1021/cr030102i 15700950

[B22] Foley T. L. Simeonov A. (2012). Targeting iron assimilation to develop new antibacterials. Expert Opin. Drug Discov. 7, 831–847. doi: 10.1517/17460441.2012.708335 22812521 PMC3434712

[B23] Fonseca É.L.d. Freitas F. Amorim J. Vicente A. C. P. (2008). Detection of new *arr-4* and *arr-5* gene cassettes in clinical *Pseudomonas aeruginosa* and *Klebsiella pneumoniae* strains from Brazil. Antimicrobial Agents Chemotherapy 52, 1865–1867. doi: 10.1128/AAC.00017-08 18299416 PMC2346643

[B24] Franke S. Grass G. Rensing C. Nies Dietrich H. (2003). Molecular analysis of the copper-transporting efflux system CusCFBA of *Escherichia coli* . J. Bacteriology 185, 3804–3812. doi: 10.1128/JB.185.13.3804-3812.2003 PMC161567 12813074

[B25] Fuster B. Salvador C. Tormo N. García-González N. Gimeno C. González-Candelas F. (2020). Molecular epidemiology and drug-resistance mechanisms in carbapenem-resistant *Klebsiella pneumoniae* isolated in patients from a tertiary hospital in Valencia, Spain. J. Global Antimicrobial Resistance 22, 718–725. doi: 10.1016/j.jgar.2020.05.002 32446938

[B26] Gomes C. Martínez-Puchol S. Palma N. Horna G. Ruiz-Roldán L. Pons M. J.. (2017). Macrolide resistance mechanisms in *Enterobacteriaceae*: Focus on azithromycin. Crit. Rev. Microbiol. 43, 1–30. doi: 10.3109/1040841X.2015.1136261 27786586

[B27] Goodman K. Simner P. Tamma P. Milstone A. (2016). Infection control implications of heterogeneous resistance mechanisms in carbapenem-resistant *Enterobacteriaceae* (CRE). Expert Rev. Anti-Infective Ther. 14, 95–108. doi: 10.1586/14787210.2016.1106940 26535959

[B28] Gündoğdu A. Long Y. B. Vollmerhausen T. L. Katouli M. (2011). Antimicrobial resistance and distribution of *sul* genes and integron-associated *intI* genes among uropathogenic *Escherichia coli* in Queensland, Australia. J. Med. Microbiol. 60, 1633–1642. doi: 10.1099/jmm.0.034140-0 21737542

[B29] Hao M. Shi X. Lv J. Niu S. Cheng S. Du H.. (2020). *In vitro* activity of apramycin against carbapenem-resistant and hypervirulent *Klebsiella pneumoniae* isolates. Front. Microbiol. 11, 425. doi: 10.3389/fmicb.2020.00425 32231657 PMC7083131

[B30] Heng P. Y. Sulong A. Wong K. K. (2019). Molecular detection of *Enterobacteriaceae* isolates producing *bla* _OXA-48_ and b*la* _OXA-181_ genes: A single centre study. Malaysian J. Pathol. 41, 139–148. 31427549

[B31] Hovan M. R. Narayanan N. Cedarbaum V. Bhowmick T. Kirn T. J. (2021). Comparing mortality in patients with carbapenemase-producing carbapenem resistant Enterobacterales and non-carbapenemase-producing carbapenem resistant Enterobacterales bacteremia. Diagn. Microbiol. Infect. Dis. 101, 115505. doi: 10.1016/j.diagmicrobio.2021.115505 34399381

[B32] Imran M. Shakoori F. R. Zulfiqar S. Khan A. T.-A. Shakoori A. R. (2024). Copper stress-induced transcriptional regulatory protein CusR also regulates silver efflux in *Klebsiella pneumoniae* KW. Pakistan J. Zoology, 1–11. doi: 10.17582/journal.pjz/20240102190224

[B33] Jolley K. A. Bray J. E. Maiden M. C. J. (2018). Open-access bacterial population genomics: BIGSdb software, the PubMLST.org website and their applications. Wellcome Open Res. 3, 124. doi: 10.12688/wellcomeopenres 30345391 PMC6192448

[B34] Jones A. K. Ranjitkar S. Lopez S. Li C. Blais J. Reck F.. (2018). Impact of inducible *bla* _DHA-1_ on susceptibility of *Klebsiella pneumoniae* clinical isolates to LYS228 and identification of chromosomal *mpl* and *ampD* mutations mediating upregulation of plasmid-borne *bla* _DHA-1_ expression. Antimicrobial Agents Chemotherapy 62(10), e01202–e01218. doi: 10.1128/AAC.01202-18 30061296 PMC6153798

[B35] Kaczmarek F. M. Dib-Hajj F. Shang W. Gootz T. D. (2006). High-level carbapenem resistance in a *Klebsiella pneumoniae* clinical isolate is due to the combination of *bla*ACT-1 β-lactamase production, porin ompK35/36 insertional inactivation, and down-regulation of the phosphate transport porin *Pho*E. Antimicrobial Agents Chemotherapy 50, 3396–3406. doi: 10.1128/AAC.00285-06 17005822 PMC1610099

[B36] Karp P. D. Billington R. Caspi R. Fulcher C. A. Latendresse M. Kothari A.. (2019). The BioCyc collection of microbial genomes and metabolic pathways. Briefings Bioinf. 20, 1085–1093. doi: 10.1093/bib/bbx085 PMC6781571 29447345

[B37] Katz L. Ashley G. W. (2005). Translation and protein synthesis: Macrolides. Chem. Rev. 105, 499–528. doi: 10.1021/cr030107f 15700954

[B38] Khalid T. W. B. M. Ghaima K. K. (2022). Effect the natural efflux pump inhibitor (Berberine) in multidrug resistant *Kleibsiella pneumoniae* isolated from urinary tract infections in several Baghdad hospitals. Egyptian J. Hosp. Med. 89, 6882–6888. doi: 10.21608/ejhm.2022.271906

[B39] Kim E.-H. Nies D. H. McEvoy M. M. Rensing C. (2011). Switch or funnel: How RND-type transport systems control periplasmic metal homeostasis. J. Bacteriology 193, 2381–2387. doi: 10.1128/JB.01323-10 PMC3133179 21398536

[B40] Kong Z. X. Karunakaran N. Abdul Jabar K. Sri La S. Sri Ponnampalavanar Chong C. W. Teh C. S. J. (2022). A retrospective study on molecular epidemiology trends of carbapenem resistant *Enterobacteriaceae* in a teaching hospital in Malaysia. PeerJ 10, e12830. doi: 10.7717/peerj.12830 35223201 PMC8877335

[B41] Krause K. M. Serio A. W. Kane T. R. Connolly L. E. (2016). Aminoglycosides: an overview. Cold Spring Harbor Perspect. Med. 6(6), a027029. doi: 10.1101/cshperspect.a027029 PMC4888811 27252397

[B42] Larsen M. V. Cosentino S. Rasmussen S. Friis C. Hasman H. Marvig R. L.. (2012). Multilocus sequence typing of total-genome-sequenced bacteria. J. Clin. Microbiol. 50, 1355–1361. doi: 10.1128/JCM.06094-11 22238442 PMC3318499

[B43] Lau M. Y. Teng F. E. Chua K. H. Ponnampalavanar S. Chong C. W. Abdul Jabar K.. (2021). Molecular characterization of carbapenem resistant *Klebsiella pneumoniae* in Malaysia hospital. Pathogens 10, 279. doi: 10.3390/pathogens10030279 33801250 PMC8001961

[B44] Lee M. Choi T.-J. (2020). Species transferability of *Klebsiella pneumoniae* carbapenemase-2 isolated from a high-risk clone of *Escherichia coli* ST410. J. Microbiol. Biotechnol. 30, 974–981. doi: 10.4014/jmb.1912.12049 32522962 PMC9728272

[B45] Lee C.-R. Lee J. H. Park K. S. Kim Y. B. Jeong B. C. Lee S. H. (2016). Global dissemination of carbapenemase-producing *Klebsiella pneumoniae*: Epidemiology, genetic context, treatment options, and detection methods. Front. Microbiol. 7, 895. doi: 10.3389/fmicb.2016.00895 27379038 PMC4904035

[B46] Lee Y. Q. Sri La S. Sri Ponnampalavanar Chong C. W. Karunakaran R. Vellasamy K. M. Abdul Jabar K.. (2022). Characterisation of non-carbapenemase-producing carbapenem-resistant *Klebsiella pneumoniae* based on their clinical and molecular profile in Malaysia. Antibiotics 11, 1670. doi: 10.3390/antibiotics11111670 36421313 PMC9686620

[B47] Lee N.-Y. Tsai C.-S. Syue L.-S. Chen P.-L. Li C.-W. Li M.-C.. (2020). Treatment outcome of bacteremia due to non–carbapenemase-producing carbapenem-resistant *Klebsiella pneumoniae* bacteremia: Role of carbapenem combination therapy. Clin. Ther. 42, e33–e44. doi: 10.1016/j.clinthera.2020.01.004 32061374

[B48] Liu X. Gong L. Liu E. Li C. Wang Y. Liang J. (2024). Characterization of the disinfectant resistance genes *qacEΔ1* and *cepA* in carbapenem-resistant *Klebsiella pneumoniae* isolates. Am. J. Trop. Med. Hygiene 110, 136–141. doi: 10.4269/ajtmh.23-0247 PMC10793004 38081061

[B49] Loftin I. R. Franke S. Roberts S. A. Weichsel A. Héroux A. Montfort W. R.. (2005). A novel copper-binding fold for the periplasmic copper resistance protein CusF. Biochemistry 44, 10533–10540. doi: 10.1021/bi050827b 16060662

[B50] Low Y. M. Yap P. S. X. Jabar K. A. Ponnampalavanar S. Karunakaran R. Velayuthan R.. (2017). The emergence of carbapenem resistant *Klebsiella pneumoniae* in Malaysia: Correlation between microbiological trends with host characteristics and clinical factors. Antimicrobial Resistance Infection Control 6, 5. doi: 10.1186/s13756-016-0164-x 28074126 PMC5219686

[B51] Mammina C. Bonura C. Aleo A. Fasciana T. Brunelli T. Pesavento G.. (2012). Sequence type 101 (ST101) as the predominant carbapenem-non-susceptible *Klebsiella pneumoniae* clone in an acute general hospital in Italy. Int. J. Antimicrobial Agents 39, 543–545. doi: 10.1016/j.ijantimicag.2012.02.012 22534506

[B52] Marimuthu K. Ng O. T. Cherng B. P. Z. Fong R. K. C. Pada S. K. De P. P.. (2019). Antecedent carbapenem exposure as a risk factor for non-carbapenemase-producing carbapenem-resistant *Enterobacteriaceae* and carbapenemase-producing *Enterobacteriaceae* . Antimicrobial Agents Chemotherapy 63, e00845–e00819. doi: 10.1128/AAC.00845-19 31383670 PMC6761519

[B53] Mohamed N. Said H. Hussin H. Abdul Rahman N. Hashim R. (2018). Carbapenem-resistant *enterobactericeae*: clinico-epidemiological perspective. Trop. Biomedicine 35, 300–307. 33601804

[B54] Morgado S. Fonseca É. Vicente A. C. (2021). Genomic epidemiology of rifampicin ADP-ribosyltransferase (Arr) in the Bacteria domain. Sci. Rep. 11, 19775. doi: 10.1038/s41598-021-99255-3 34611248 PMC8492726

[B55] Nicolas-Chanoine M.-H. Mayer N. Guyot K. Dumont E. Pagès J.-M. (2018). Interplay between membrane permeability and enzymatic barrier leads to antibiotic-dependent resistance in *Klebsiella pneumoniae* . Front. Microbiol. 9, 1422. doi: 10.3389/fmicb.2018.01422 30008709 PMC6034560

[B56] Orsi G. B. Bencardino A. Vena A. Carattoli A. Venditti C. Falcone M.. (2013). Patient risk factors for outer membrane permeability and KPC-producing carbapenem-resistant *Klebsiella pneumoniae* isolation: Results of a double case-control study. Infection 41, 61–67. doi: 10.1007/s15010-012-0354-2 23070604

[B57] Overbeek R. Olson R. Pusch G. D. Olsen G. J. Davis J. J. Disz T.. (2014). The SEED and the Rapid Annotation of microbial genomes using Subsystems Technology (RAST). Nucleic Acids Res. 42, D206–D214. doi: 10.1093/nar/gkt1226 24293654 PMC3965101

[B58] Padilla E. Llobet E. Doménech-Sánchez A. Martínez-Martínez L. Bengoechea J. A. Albertí S. (2010). *Klebsiella pneumoniae* AcrAB efflux pump contributes to antimicrobial resistance and virulence. Antimicrobial Agents Chemotherapy 54, 177–183. doi: 10.1128/AAC.00715-09 19858254 PMC2798511

[B59] Paulsen I. T. Littlejohn T. G. Rådström P. Sundström L. Sköld O. Swedberg G.. (1993). The 3’ conserved segment of integrons contains a gene associated with multidrug resistance to antiseptics and disinfectants. Antimicrobial Agents Chemotherapy 37, 761–768. doi: 10.1128/AAC.37.4.761 8494372 PMC187754

[B60] Perron K. Caille O. Rossier C. Van Delden C. Dumas J.-L. Köhler T. (2004). CzcR-CzcS, a two-component system involved in heavy metal and carbapenem resistance in *Pseudomonas aeruginosa* . J. Biol. Chem. 279, 8761–8768. doi: 10.1074/jbc.M312080200 14679195

[B61] Randall C. P. Gupta A. Jackson N. Busse D. O’Neill A. J. (2015). Silver resistance in Gram-negative bacteria: A dissection of endogenous and exogenous mechanisms. J. Antimicrobial Chemotherapy 70, 1037–1046. doi: 10.1093/jac/dku523 PMC4356207 25567964

[B62] Richards M. Cruickshank M. Cheng A. Gandossi S. Quoyle C. Stuart R.. (2017). Recommendations for the control of carbapenemase-producing *Enterobacteriaceae* (CPE): A guide for acute care health facilities: Australian Commission on Safety and Quality in Health Care. Infection Dis. Health 22, 159–186. doi: 10.1016/j.idh.2017.09.001

[B63] Rodrigue A. Effantin G. Mandrand-Berthelot M.-A. (2005). Identification of *rcnA* (*yohM*), a nickel and cobalt resistance gene in *Escherichia coli* . J. Bacteriology 187, 2912–2916. doi: 10.1128/JB.187.8.2912-2916.2005 PMC1070376 15805538

[B64] Rosas N. C. Wilksch J. Barber J. Li J. Wang Y. Sun Z.. (2023). The evolutionary mechanism of non-carbapenemase carbapenem-resistant phenotypes in Klebsiella spp. Elife 12, e83107. doi: 10.7554/eLife.83107.sa2 37410078 PMC10325707

[B65] Ruiz J. (2019). Transferable mechanisms of quinolone resistance from 1998 onward. Clin. Microbiol. Rev. 32(4), e00007–e00019. doi: 10.1128/cmr.00007-19 31413045 PMC6730498

[B66] Saw H. T. Webber M. A. Mushtaq S. Woodford N. Piddock L. J. (2016). Inactivation or inhibition of AcrAB-TolC increases resistance of carbapenemase-producing *Enterobacteriaceae* to carbapenems. J. Antimicrobial Chemotherapy 71, 1510–1519. doi: 10.1093/jac/dkw028 26945714

[B67] Schwanbeck J. Bohne W. Hasdemir U. Groß U. Pfeifer Y. Bunk B.. (2021). Detection of a new resistance-mediating plasmid chimera in a *bla* _OXA-48_-positive *Klebsiella pneumoniae* strain at a German University Hospital. Microorganisms 9, 720. doi: 10.3390/microorganisms9040720 33807212 PMC8066831

[B68] Shin H. W. Lim J. Kim S. Kim J. Kwon G. C. Koo S. H. (2015). Characterization of trimethoprim-sulfamethoxazole resistance genes and their relatedness to class 1 integron and insertion sequence common region in Gram-negative bacilli. J. Microbiol. Biotechnol. 25, 137–142. doi: 10.4014/jmb.1409.09041 25348695

[B69] Su C.-F. Chuang C. Lin Y.-T. Chan Y.-J. Lin J.-C. Lu P.-L.. (2018). Treatment outcome of non-carbapenemase-producing carbapenem-resistant *Klebsiella pneumoniae* infections: A multicenter study in Taiwan. Eur. J. Clin. Microbiol. Infect. Dis. 37, 651–659. doi: 10.1007/s10096-017-3156-8 29238934

[B70] Tacconelli E. Carrara E. Savoldi A. Harbarth S. Mendelson M. Monnet D. L.. (2018). Discovery, research, and development of new antibiotics: The WHO priority list of antibiotic-resistant bacteria and tuberculosis. Lancet Infect. Dis. 18, 318–327. doi: 10.1016/S1473-3099(17)30753-3 29276051

[B71] Tamma P. D. Goodman K. E. Harris A. D. Tekle T. Roberts A. Taiwo A.. (2017). Comparing the outcomes of patients with carbapenemase-producing and non-carbapenemase-producing carbapenem-resistant *Enterobacteriaceae* bacteremia. Clin. Infect. Dis. 64, 257–264. doi: 10.1093/cid/ciw741 28013264 PMC5241781

[B72] Tamura K. Stecher G. Kumar S. (2021). MEGA11: molecular evolutionary genetics analysis version 11. Mol. Biol. Evol. 38, 3022–3027. doi: 10.1093/molbev/msab120 33892491 PMC8233496

[B73] Westblade L. (2018). Burning question - When should clinical microbiology laboratories perform carbapenemase detection tests? Clin. Lab. Standards Institute (CLSI). 3(1), 12–14. Available at: https://clsi.org/about/blog/when-should-clinical-microbiology-laboratories-perform-carbapenemase-detection-tests/.

[B74] Wyres K. L. Lam M. M. Holt K. E. (2020). Population genomics of *Klebsiella pneumoniae* . Nat. Rev. Microbiol. 18, 344–359. doi: 10.1038/s41579-019-0315-1 32055025

[B75] Yeow K. K. (2020). Epidemiology study of carbapenam-resistant *Enterobacteriaceae* (CRE): A 30-months experience at a tertiary centre in northern Malaysia. Int. J. Infect. Dis. 101, 69. doi: 10.1016/j.ijid.2020.09.211

[B76] Zaidah A. R. Mohammad N. I. Suraiya S. Harun A. (2017). High burden of carbapenem-resistant *Enterobacteriaceae* (CRE) fecal carriage at a teaching hospital: Cost-effectiveness of screening in low-resource setting. Antimicrobial Resistance Infection Control 6, 1–6. doi: 10.1186/s13756-017-0200-5 PMC5414356 28473912

[B77] Zankari E. Hasman H. Cosentino S. Vestergaard M. Rasmussen S. Lund O.. (2012). Identification of acquired antimicrobial resistance genes. J. Antimicrobial Chemotherapy 67, 2640–2644. doi: 10.1093/jac/dks261 PMC3468078 22782487

[B78] Zawawi R. D. Ramli R. Sidik T. M. I. T. A. Naina-Mohamed I. Loon L. C. (2021). Clinical characteristics and risk factors of carbapenem-resistant *Enterobacteriaceae*: A case-control study in a tertiary hospital in Malaysia. Malaysian J. Med. Health Sci. 17(4), 189–195.

[B79] Zhang Y. Jiang X. Wang Y. Li G. Tian Y. Liu H.. (2014). Contribution of β-lactamases and porin proteins ompK35 and ompK36 to carbapenem resistance in clinical isolates of *KPC*-2-producing *Klebsiella pneumoniae* . Antimicrobial Agents Chemotherapy 58, 1214–1217. doi: 10.1128/AAC.02045-12 24277031 PMC3910872

[B80] Zhu M. Valdebenito M. Winkelmann G. Hantke K. (2005). Functions of the siderophore esterases IroD and IroE in iron-salmochelin utilization. Microbiology 151, 2363–2372. doi: 10.1099/mic.0.27888-0 16000726

[B81] Zulfiqar S. Shakoori A. R. (2012). Molecular characterization, metal uptake and copper induced transcriptional activation of efflux determinants in copper resistant isolates of *Klebsiella pneumoniae* . Gene 510, 32–38. doi: 10.1016/j.gene.2012.08.035 22960400

