# Supplementary material for: Investigation on the mechanisms of carbapenem resistance among the non-carbapenemase-producing carbapenem-resistant Klebsiella pneumoniae
Source: Front Cell Infect Microbiol. 2024 Sep 18;14:1464816. doi: 10.3389/fcimb.2024.1464816 (PMC11445613; doi:10.3389/fcimb.2024.1464816)
Supplement: Supplementary file 1 [file Table1.docx]

Supplementary Materials

# Supplementary Data

Table S1. MIC reduction assay of carbapenem and ciprofloxacin in the absence and presence of the efflux pump inhibitor PAβN.

| **Isolates** | **1801-2** | **1804-1** | **1805-11** | **1805-12** |
| --- | --- | --- | --- | --- |
| IPM | S | S | R | R |
| IPM | 0.5 | 1 | 4 | 32 |
| IPM + PAβN | - | - | 4 | 32 |
| MEM | S | S | R | R |
| MEM | 0.125 | 0.5 | 16 | 8 |
| MEM + PAβN | - | - | 16 | 32 |
| ETP | R | R | R | R |
| ETP | 16 | 8 | 128 | 128 |
| ETP + PAβN | 128 | 32 | 256 | 128 |
| CIP | R | R | R | R |
| CIP | >512 | 8 | 32 | 8 |
| CIP + PAβN | 256* | 2* | 8* | 2* |

^*^ Isolates with a ≥ 4-fold decrease in MIC in the presence of PAβN (26.3 µg/ml).

Antimicrobial agents: Ertapenem (ETP), imipenem (IPM), meropenem (MEM), or ciprofloxacin (CIP). The “R” denotes resistant, and “S” denotes susceptible.

The symbol “-” denotes not applicable.

*Note.* Adapted from “Characterisation of non-carbapenemase-producing carbapenem-resistant *Klebsiella pneumoniae* based on their clinical and molecular profile in Malaysia”, by Lee *et al*., 2022, *Antibiotics*, 11(11): 1670 (<https://doi.org/10.3390/antibiotics11111670>). CC BY.

Table S2. Enzymes in the peptidoglycan biosynthesis superpathways of *Klebsiella pneumoniae*.

| **Gene** | **Enzyme** | **Accession number** | **Polymorphism** |
| --- | --- | --- | --- |
|  |  |  |  |
| *Peptidoglycan biosynthesis I pathway* | |  |  |
|  |  |  |  |
| *murA* | UDP-N-acetylglucosamine 1-carboxyvinyltransferase | WP_002918382.1 | No |
| *murB* | UDP-N-acetylmuramate dehydrogenase | WP_002883510.1 | Yes |
| *murC* | UDP-N-acetylmuramate--L-alanine ligase | WP_004178595.1 | Yes |
| *A8C11_RS07165* | Aspartate/glutamate racemase | WP_004174519.1 | Yes |
| *murI* | Glutamate racemase | WP_002883026.1 | Yes |
| *murD* | UDP-N-acetylmuramoyl-L-alanine--D-glutamate ligase | WP_004147075.1 | Yes |
| *murE* | UDP-N-acetylmuramoyl-L-alanyl-D-glutamate--2, 6-diaminopimelate ligase | WP_004147071.1 | Yes |
| *A8C11_RS15935* | D-alanine--D-alanine ligase | WP_002888623.1 | No |
| *ddlA* | D-alanine--D-alanine ligase | WP_004177279.1 | Yes |
| *murF* | UDP-N-acetylmuramoyl-tripeptide--D-alanyl-D-alanine ligase | WP_004177411.1 | No |
| *mraY* | Phospho-N-acetylmuramoyl-pentapeptide-transferase | WP_002888562.1 | No |
| *murG* | Undecaprenyldiphospho-muramoylpentapeptide beta-N-acetylglucosaminyltransferase | WP_002888566.1 | No |
|  |  |  |  |
| *Peptidoglycan maturation pathway* | |  |  |
|  |  |  |  |
| *pbpC* | Peptidoglycan glycosyltransferase PbpC | WP_004180894.1 | Yes |
| *mtgA* | Monofunctional biosynthetic peptidoglycan transglycosylase | WP_004144927.1 | Yes |
| *mrcA* | Peptidoglycan glycosyltransferase/peptidoglycan DD-transpeptidase MrcA | WP_004181454.1 | Yes |
| *mrdB* | Peptidoglycan glycosyltransferase MrdB | WP_002894613.1 | No |
| *dacD* | Serine-type D-Ala-D-Ala carboxypeptidase DacD | WP_004196295.1 | Yes |
| *dacB* | Serine-type D-Ala-D-Ala carboxypeptidase | WP_004149937.1 | No |
| *dacA* | D-alanyl-D-alanine carboxypeptidase DacA | WP_002894539.1 | No |
| *mrdA* | Peptidoglycan DD-transpeptidase MrdA | WP_002894617.1 | No |
| *dacC* | Serine-type D-Ala-D-Ala carboxypeptidase | WP_004191175.1 | Yes |
| *ftsI* | Peptidoglycan glycosyltransferase FtsI | WP_002910924.1 | No |
| *mrdA* | Penicillin-binding protein 2 | WP_004180024.1 | Yes |
| *ampH* | D-alanyl-D-alanine- carboxypeptidase/endopeptidase AmpH | WP_004178722.1 | Yes |
| *ldtA* | L,D-transpeptidase | WP_004180454.1 | Yes |
| *ldtB* | L,D-transpeptidase | WP_002895871.1 | No |
| *ldtD* | L,D-transpeptidase | WP_002898195.1 | Yes |
| *mepS* | Bifunctional murein DD-endopeptidase/murein LD-carboxypeptidase | WP_002912967.1 | No |
| *ldcA* | Muramoyltetrapeptide carboxypeptidase | WP_002910883.1 | Yes |
